# Empowering nursing students in microbiology: the impact of a PhotoVoice learning activity – a mixed-methods study

**DOI:** 10.1099/acmi.0.001265.v3

**Published:** 2026-11-13

**Authors:** Chun Sing Lam, Ho Lung Wong, Edmond Pui Hang Choi

**Affiliations:** 1 School of Nursing, The University of Hong Kong, Hong Kong, Hong Kong, SAR

**Keywords:** engagement, learning empowerment, microbiology class, photovoice, satsifaction

## Abstract

Medical microbiology is a core component of nursing education but is often perceived as challenging and disconnected from clinical practice. Active learning strategies, such as PhotoVoice, may enhance engagement and bridge this gap yet evidence for their effectiveness in nursing microbiology education is limited. To evaluate the impact of a PhotoVoice learning activity on pre-registration nursing students’ engagement, learning empowerment and satisfaction in a microbiology class. An observational, mixed-methods study with a single-group pretest–posttest design and qualitative group interviews was conducted in two pre-registration nursing programmes (bachelor’s and master’s) at a university in Hong Kong. A total of 207 nursing students (188 bachelor’s, 19 master’s) completed pre- and post-intervention questionnaires; 128 students participated in group interviews. Both bachelor’s and master’s students demonstrated statistically significant improvements in engagement following the PhotoVoice activity (*P*<0.001, *d*=0.94 for bachelor’s; *P*=0.003, *d*=0.79 for master’s) and learning empowerment (*P*<0.001, *d*=1.08 for bachelor’s; *P*=0.002, *d*=0.84 for master’s) following the activity. Bachelor’s students’ satisfaction also improved (*P*<0.001, *d*=0.75). Qualitative analysis identified five core themes: enhanced engagement, active and experiential learning, intellectual stimulation, authentic contexts and innovative pedagogy. The PhotoVoice activity is correlated with enhanced engagement, empowerment and satisfaction, supporting its integration as an active learning strategy in nursing microbiology education.

## Data Summary

All data associated with this work is reported within the article and supplementary files.

## Introduction

Medical microbiology, a foundational life science subject within the nursing curriculum, is widely recognized as a core component of nursing education [1]. It provides essential knowledge for safe and effective clinical nursing practice by forming the scientific basis for understanding infection control, disease transmission and their impact on patient care. Given its clinical relevance, nurses routinely apply medical microbiology knowledge in tasks such as using aseptic techniques, preventing hospital-acquired infections, administering antibiotics, collecting and handling specimens and educating patients about infection risks [2].

However, nursing students frequently report that medical microbiology is a challenging subject and often struggle to stay motivated [3]. For instance, a qualitative study exploring nursing students’ learning experience in microbiology in South Korea found that the unfamiliar content posed a significant barrier to learning. Some students even questioned the necessity of studying microbiology and expressed difficulty in understanding the course material [3]. Another qualitative study involving practising nurses supports these findings, with one participant noting that she did not understand the clinical significance of microbiology during her training [4]. These learning challenges may contribute to inadequate microbiology knowledge. A survey study involving 523 pre-registration nursing students from 23 universities in the United Kingdom revealed notable gaps in students’ understanding of basic sciences – particularly microbiology – relevant to antimicrobial stewardship activities commonly performed by nurses [1].

These knowledge gaps are not limited to nursing students but are also evident among practising nurses. A study conducted in South Korea reported that nurses' knowledge of clinical microbiology was at a moderate level, with an average score of 5.25 out of 10. The lowest-scoring areas included topics such as the characteristics of bacteria (e.g. *Neisseria* species and anaerobic bacteria) and the structure of viruses. In contrast, nurses demonstrated higher levels of knowledge in areas more directly related to clinical practice, particularly infection control measures such as disinfection and sterilization [5]. A study conducted in the USA specifically assessed health professionals’ (registered nurses and registered dietitians) knowledge and understanding of *Listeria monocytogenes*. The findings revealed that, compared to dietitians, nurses had a significantly lower level of awareness, understanding and clinical knowledge related to *L. monocytogenes* infection [6].

Given the identified gaps in microbiology knowledge among both nursing students and practising nurses, it has been recommended that pre-registration nursing education programmes place greater emphasis on strengthening students’ understanding of basic medical microbiology [1]. Enhancing this foundational knowledge can help students recognize the presence of unseen micro-organisms in clinical environments and understand how to prevent the spread of infection [7]. In terms of preferred teaching methods for learning medical microbiology, the United Kingdom study found that students favoured face-to-face instruction, problem-based learning and blended learning approaches that combine classroom teaching with online activities. In contrast, online-only learning, stand-alone e-learning modules and student-led presentations were perceived as less effective [1].

Didactic lectures remain a widely used teaching method in universities, particularly in large classes and resource-limited settings. However, there is a growing shift towards integrating more interactive and student-centred approaches, as traditional lectures are increasingly viewed as less effective in promoting student engagement and meaningful learning [8]. This teacher-centred mode of instruction often positions students as passive recipients of information, with limited opportunities for real-world application [9]. As a result, relying solely on didactic lectures without incorporating active learning strategies may hinder students’ development of creativity, critical thinking and motivation to learn.

According to a systematic review that conducted an in-depth qualitative analysis of 161 definitions of active learning, three key themes emerged [10]. First, active learning is grounded in student-centred constructivist theory; second, it promotes higher-order thinking and deep learning; and third, it is characterized by instructional strategies that involve activity, participation and engagement [10]. The effectiveness of active learning on various student outcomes is well-documented. For example, a study conducted in the USA among undergraduate students enrolled in biology modules found that implementing active learning strategies within a flipped classroom model enhanced students’ critical thinking skills [11]. Another US-based study, conducted among science majors in an introductory biology laboratory course, reported that active learning approaches increased students’ interest, creativity and motivation to prepare [12]. Moreover, it created a challenging learning environment that encouraged the sharing of diverse perspectives and fostered a sense of community among learners [12].

In the context of nursing education, a study in Morocco found that active learning strategies improved nursing students’ communication skills, attitudes, teamwork and stress management competencies [13]. Similarly, focus group interviews with nurse educators in Bosnia and Herzegovina revealed that the implementation of active learning methods in undergraduate nursing programmes had a positive effect on the development of students’ communication and critical thinking skills [14]. These findings highlight the growing body of evidence supporting the integration of active learning strategies into pre-registration nursing curricula to supplement traditional didactic lectures and enhance student learning outcomes.

PhotoVoice, first introduced by Wang and Burris [15], originated as a participatory action research method aimed at making knowledge development accessible by involving communities throughout the research process [16, 17]. In its original form, known as ‘photo novella’, it was used to document the daily struggles of women in Yunnan Province, China [15]. In PhotoVoice, participants use photography to capture aspects of their lives and then develop narratives around these images [18, 19]. Over time, PhotoVoice has evolved into a research-based and innovative pedagogical tool that empowers participants – including students – to express their perspectives and experiences through photography [16, 20]. As a teaching method, it integrates images with reflective discussions, enabling students to capture meaningful photographs relevant to their learning and provide commentary. The use of PhotoVoice in education has spanned a wide range of settings and disciplines, from environmental science and chemistry to design education and health sciences and across learner levels from high school to higher education [16, 21–25]. PhotoVoice has been shown to enhance engagement, self-expression, critical thinking and in-depth understanding of complex issues [26–28].

Pedagogically, PhotoVoice shifts learning from a positivistic, teacher-centred approach to a more constructivist, student-centred one, promoting deeper understanding, the relevance of content to students’ lived realities and authentic learning experiences [22, 29]. Authenticity has been shown to positively influence learning motivation, which in turn sustains student engagement [29]. By encouraging reflection and class discussion around the images produced, students can explore the personal and societal significance of their subject matter while developing collaboration and communication skills [16].

Despite its demonstrated benefits, there remains a lack of research examining the application of PhotoVoice in the microbiology modules of pre-registration nursing curricula. In this study, we aimed to explore the effect of an exploratory PhotoVoice learning activity on pre-registration nursing students’ engagement, learning autonomy and satisfaction in the microbiology classroom.

## Methodology

### Study design

This observational study employed a mixed-methods approach. A single-group pretest–posttest design was used to evaluate the effect of the PhotoVoice learning activity. In addition, qualitative feedback on the learning experience was gathered through group interviews, allowing for a deeper exploration of students’ perceptions and reflections.

### Setting, study population, sampling

The PhotoVoice learning activity was implemented as an educational innovation within microbiology courses offered in two pre-registration nursing programmes at a government-funded university in Hong Kong. These programmes included a Bachelor-level programme and an entry-to-practice Master’s programme designed for students who already hold a non-nursing bachelor’s degree and wish to become registered nurses. The microbiology course was offered in Year 2 of the Bachelor’s programme and in Year 1 of the Master’s programme. The PhotoVoice activity was embedded in the course as one of the required learning components. Therefore, all students enrolled in the courses were expected to participate as part of their coursework, and no specific inclusion or exclusion criteria were applied. However, only students who provided written informed consent were included in the research component of the study.

### Study procedures

Prior to the implementation of the PhotoVoice learning activity, all participating nursing students completed an online pre-intervention questionnaire. This was followed by a structured briefing session conducted by the project lead – a lecturer with a doctoral degree in life sciences – who introduced the concept and procedures of the intervention. During this session, students were trained in the proper use of an adenosine triphosphate (ATP) bioluminescence checker and surface sampling swabs. The lecturer also provided ethical and technical guidance on photography and data collection at public and semi-public locations on campus.

The PhotoVoice learning activity was implemented in small groups, with students randomly assigned to teams of four to five. Each group was tasked with identifying what they perceived to be the cleanest and dirtiest inanimate surface locations within the medical campus environment. After reaching consensus, groups were allowed to leave the classroom and visit their selected sites to collect samples and document findings (Fig. 1).

**Fig. 1.**
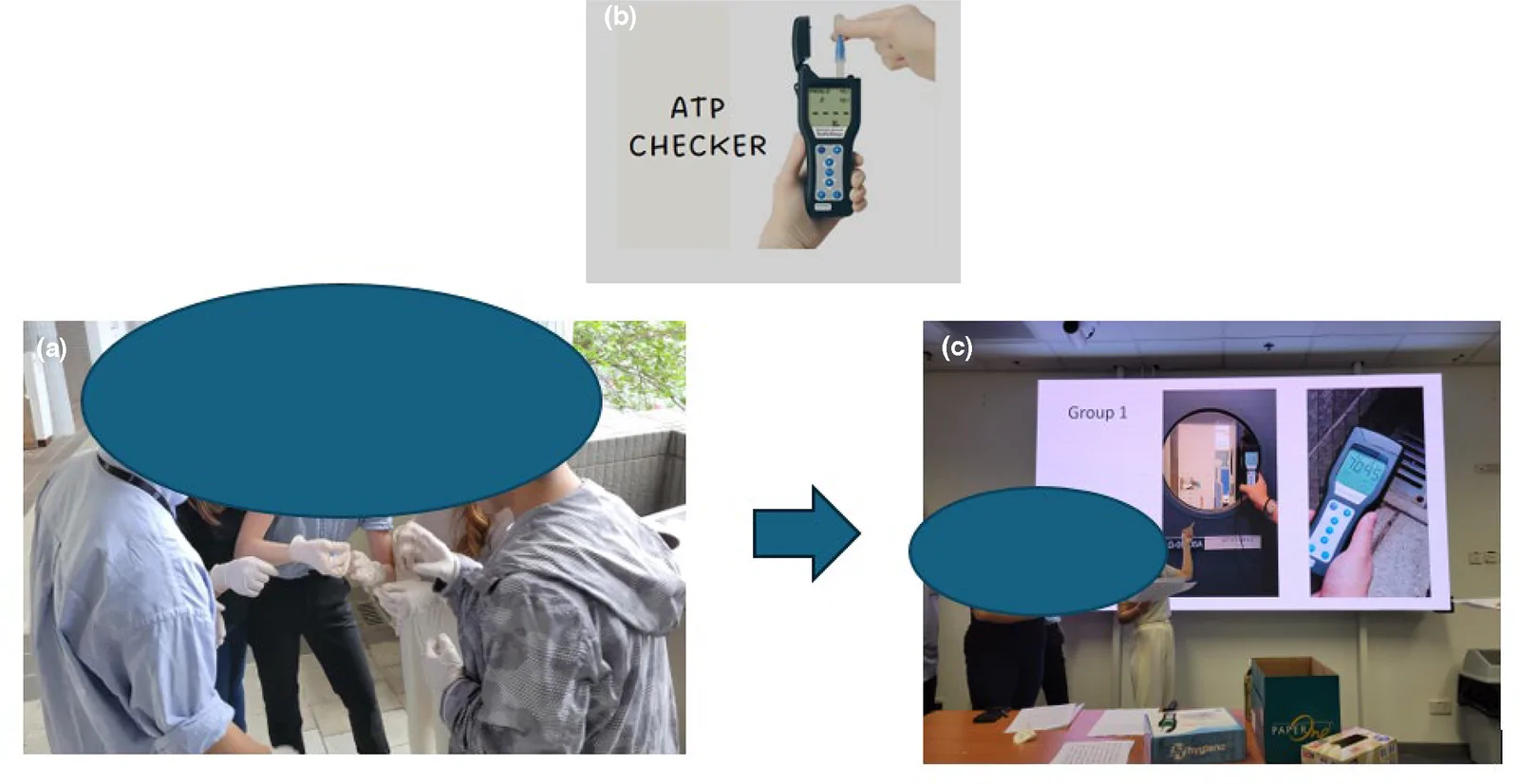
Illustration of explorative PhotoVoice activity implementation from site sampling and data record (a) with the use of ATP checker (b) and presentation at the reflective session (c).

Using sterile UltraSnap™ ATP swabs (Pack and Vac Limited, Hong Kong), students collected surface samples from each selected site (Fig. 2) . These samples were analysed immediately using the SystemSURE™ PLUS ATP bioluminescence metre (Pack and Vac Limited, Hong Kong), a portable device that measures surface cleanliness through detection of organic residue in the form of ATP. The ATP bioluminescence assay has been validated in various contexts – including schools, food industries and healthcare settings – as a rapid and reliable method for assessing environmental hygiene [30–33].

**Fig. 2.**
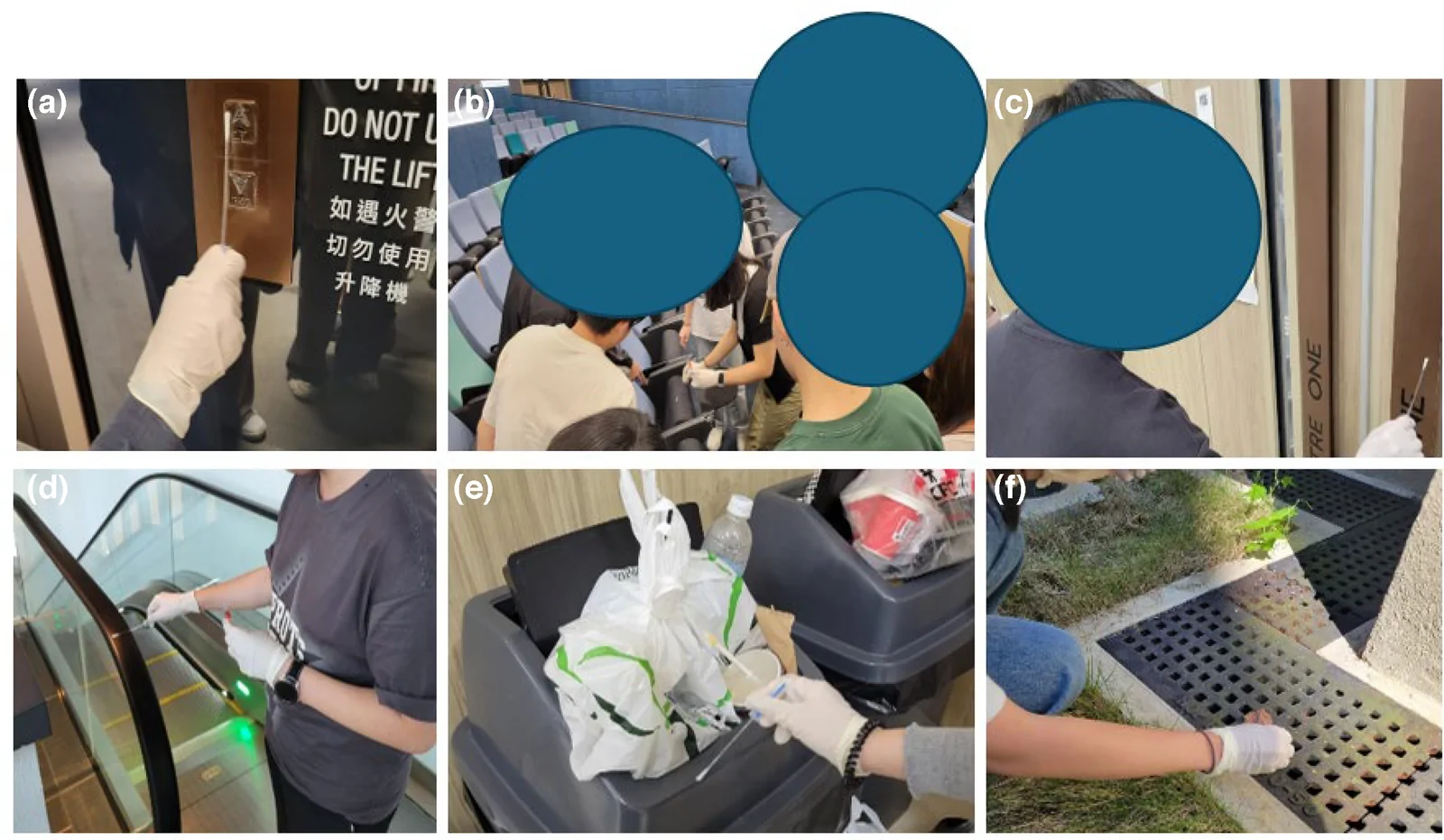
Example of selected sampling sites (a) lift button, (**b**) lecture theatre, (**c**) door handle, (**d**) escalator handle, (**e**) trash box and (f) manhole cover.

Following sample analysis, students used their mobile phones to capture photographs of the two sites (cleanest and dirtiest) along with the corresponding ATP readings. These visual data served as the foundation for the subsequent PhotoVoice reflective session, conducted in the classroom.

During the reflection session, students presented their findings to peers using the PhotoVoice framework, guided by the structured SHOWED prompts [34] (Fig. 1):

1. What do you *S*ee here?
2. What is really *H*appening here?
3. How does this relate to *O*ur lives?
4. *W*hy does this problems or situation exist?
5. How can this image *E*ducate others?
6. What can we *D*o about this problem or situation?

This structured reflection encouraged students to critically analyse their observations, connect them to clinical practice and consider broader public health implications. Immediate formative feedback was provided by the lecturer and classmates, fostering dialogue and peer learning. This learning activity was designed not only to reinforce core microbiology concepts through experiential and visual learning but also to enhance students’ critical thinking, communication skills and awareness of environmental hygiene in clinical and community settings.

After the reflective session, students were invited to complete an online post-intervention questionnaire. In addition, participants were invited to take part in group interviews to explore their experiences and reflections on the PhotoVoice learning activity. These interviews were conducted in the same groups formed during the activity and were facilitated by a trained research assistant who had no involvement in the design, delivery or evaluation of the activity. This approach was intended to encourage open discussion and reduce potential bias due to the dual role of the course lecturer as project lead.

Due to differences in class schedules between the Bachelor and Master programmes, the teaching innovation was implemented separately for each group. This approach allowed the intervention to align with the specific timetables of each cohort. To ensure consistency in delivery, the project lead conducted the intervention for both cohorts.

### Study variables and study instruments (quantitative evaluation)

#### Student engagement

Students’ engagement in the PhotoVoice learning activities was measured using the six subscales of the Student Engagement Questionnaire developed by Kember and Leung [35] at both pre- and post-intervention. These subscales include (i) critical thinking, (ii) creative thinking, (iii) self-managed learning, (iv) problem solving, (v) interpersonal skills and group work and (vi) active learning. Each subscale consists of two question items. Students rated all items on a five-point Likert scale ranging from Strongly Disagree (1) to Strongly Agree (5). Both subscale scores and the overall score were used to evaluate students’ engagement. Higher scores indicate a higher level of engagement. Additionally, at the post-intervention stage, a self-developed item was included to assess overall engagement in the PhotoVoice activities: ‘*I am fully engaged in the class with the use of the PhotoVoice activity*’. This item was also rated using the same five-point Likert scale.

#### Learning empowerment

Student empowerment was measured using selected items from the Learner Empowerment Instrument developed by Frymier *et al*. [36] at both pre- and post-intervention. The original instrument comprises 35 items across three domains: (i) impact (16 items), (ii) meaningfulness (10 items) and (iii) competence (9 items). In this study, only the most relevant items were selected based on a review by the investigation team, resulting in five items for impact, five for meaningfulness and three for competence. All items were rated on a five-point Likert scale (from zero to four). Both subscale scores and the overall score were used to evaluate students’ learning autonomy, with higher scores indicating a higher level of student empowerment.

In addition, at the post-intervention stage, a self-developed item was included to assess overall learning empowerment in the PhotoVoice activities: *‘I am provided sufficient learning empowerment with the use of the PhotoVoice activity.’* This item was also rated using the same five-point Likert scale.

#### Student satisfaction

Students’ satisfaction in the PhotoVoice learning activity was measured using selected items from the Student Outcomes Survey in Australia [37], at both pre- and post-intervention. The original survey comprises 20 items across three domains – teaching (6 items), assessment (5 items) and generic skills and learning experiences (8 items) – plus a single overall satisfaction question. For this study, the investigation team selected the most relevant items, retaining five items for teaching, two for assessment and four for generic skills and learning experiences. All items were rated on a five-point Likert scale, with values 1–5. Subscale scores and the overall satisfaction score were used to assess student satisfaction, with higher scores indicating greater satisfaction.

An additional self-developed item was included at the post-intervention stage to evaluate overall satisfaction with the PhotoVoice activity: ‘*Overall, I was satisfied with the quality of this training with the use of the PhotoVoice activity*’. This item was also rated using the same five-point Likert scale.

### Group interview (qualitative evaluation)

To complement the quantitative data, group interviews were conducted to explore participants’ experiences and perceptions of the PhotoVoice learning activity. A semi-structured format was used, guided by the following three questions:

- What was the most pleasurable aspect of your experience with the PhotoVoice activity?
- What was the most difficult or unpleasant aspect of your experience with the PhotoVoice activity?
- Compared to traditional experiments, did the PhotoVoice activity help you learn microbiology more effectively? If so, how?

Each session was audio-recorded for subsequent analysis. Participants were informed prior to the interview that their participation was voluntary and that they could withdraw at any time without penalty.

#### Data analysis

Quantitative data were analysed using descriptive statistics – means and sd – to summarize the study outcomes: student engagement, empowerment and satisfaction. For each outcome, domain-specific subscale scores and overall scale scores were calculated. To evaluate the impact of the PhotoVoice activity on study outcomes, a paired t-test was used to compare pre- and post-intervention scores, and Cohen’s *d* was calculated to determine effect sizes. Additionally, descriptive statistics, including frequency (number) and percentage, were used to summarize students’ engagement, empowerment and satisfaction with the PhotoVoice activity as assessed by the self-developed questions at post-intervention. Analyses were conducted separately for the bachelor’s and master’s programmes. All statistical analyses were performed using SPSS version 28.

The group interview data were analysed using inductive content analysis, following the steps outlined by Elo and Kyngäs [38]. This approach was selected to allow patterns and categories to emerge directly from the data, without imposing preconceived frameworks. Audio recordings from each group interview were listened to multiple times by the project lead and the trained research assistant who conducted the interviews. Detailed notes and meaning units were extracted directly from the recordings. A meaning unit was defined as a word, phrase or sentence that conveyed a distinct idea or perception related to the PhotoVoice learning activity. During the open coding phase, meaning units were examined and assigned descriptive codes that captured their essential content. Similar codes were then grouped into subcategories based on conceptual similarity. These subcategories were further abstracted into broader categories, each representing a key dimension of students’ experiences. The process was iterative and collaborative, involving regular discussions between the two analysts to reach consensus and ensure consistency in coding and interpretation.

## Results

For the original class sizes, there were 326 students in the bachelor’s programme and 27 students in the master’s programme. Of these, 188 students in the bachelor’s programme completed both the pre- and post-intervention questionnaires, whereas 19 students from the master’s programme completed the pre- and post-intervention questionnaires.

### Student engagement

Among the bachelor’s degree students, statistically significant improvements were observed across all six engagement subscales and the overall engagement score following the implementation of the PhotoVoice activity. The total engagement score increased from a pre-intervention mean of 46.90 (sd=6.95) to 53.74 (sd=6.65) post-intervention, representing a large effect size (Cohen’s *d*=0.94). Notably, the largest gains were found in critical thinking (Cohen’s *d*=0.90), creative thinking (Cohen’s *d*=0.86) and interpersonal skills and groupwork (Cohen’s *d*=0.86). In addition, at the post-intervention assessment, 183 students (97.34%) agreed or strongly agreed with the statement: ‘*I am fully engaged in the class with the use of the PhotoVoice activity*’.

Similarly, master’s degree students showed statistically significant improvements in overall engagement and all six subscales, despite the smaller sample size. The most substantial effect sizes were observed for creative thinking (Cohen’s *d*=0.81), critical thinking (Cohen’s *d*=0.77) and interpersonal skills and groupwork (Cohen’s *d*=0.71), with the total engagement score increasing from 49.84 (sd=4.41) to 54.05 (sd=5.98; Cohen’s *d*=0.79). Additionally, at the post-intervention assessment, 18 students (94.73%) agreed or strongly agreed with the same engagement statement. Table 1 presents the detailed results of student engagement and Fig. 3 illustrates the comparative changes in engagement levels before and after the intervention.

**Table 1.** Effect of PhotoVoice learning activity on student engagement

Pre-intervention (mean±sd)	Post-intervention (mean±sd)	*P*-value	Cohen’s *d* effect size
**Students in the bachelor’s programme (*n*=188)**				
Critical thinking	7.79±1.23	8.94±1.16	<0.001	0.90
Creative thinking	7.76±1.28	8.95±1.16	<0.001	0.86
Self-managed learning	7.84±1.32	8.94±1.21	<0.001	0.78
Problem-solving	7.80±1.30	8.89±1.25	<0.001	0.78
Interpersonal skills and groupwork	7.71±1.40	8.86±1.28	<0.001	0.86
Active learning	8.01±1.33	9.16±1.08	<0.001	0.80
Total	46.90±6.95	53.74±6.65	<0.001	0.94
**Students in the master’s programme (*n*=19)**				
Critical thinking	8.26±0.87	9.05±1.03	0.004	0.77
Creative thinking	8.21±0.79	9.00±1.11	0.002	0.81
Self-managed learning	8.26±1.10	9.05±1.03	0.007	0.70
Problem-solving	8.26±0.87	8.95±0.97	0.015	0.62
Interpersonal skills and groupwork	8.26±0.65	9.00±1.00	0.007	0.71
Active learning	8.58±0.96	9.00±1.00	0.057	0.47
Total	49.84±4.41	54.05±5.98	0.003	0.79

**Fig. 3.**
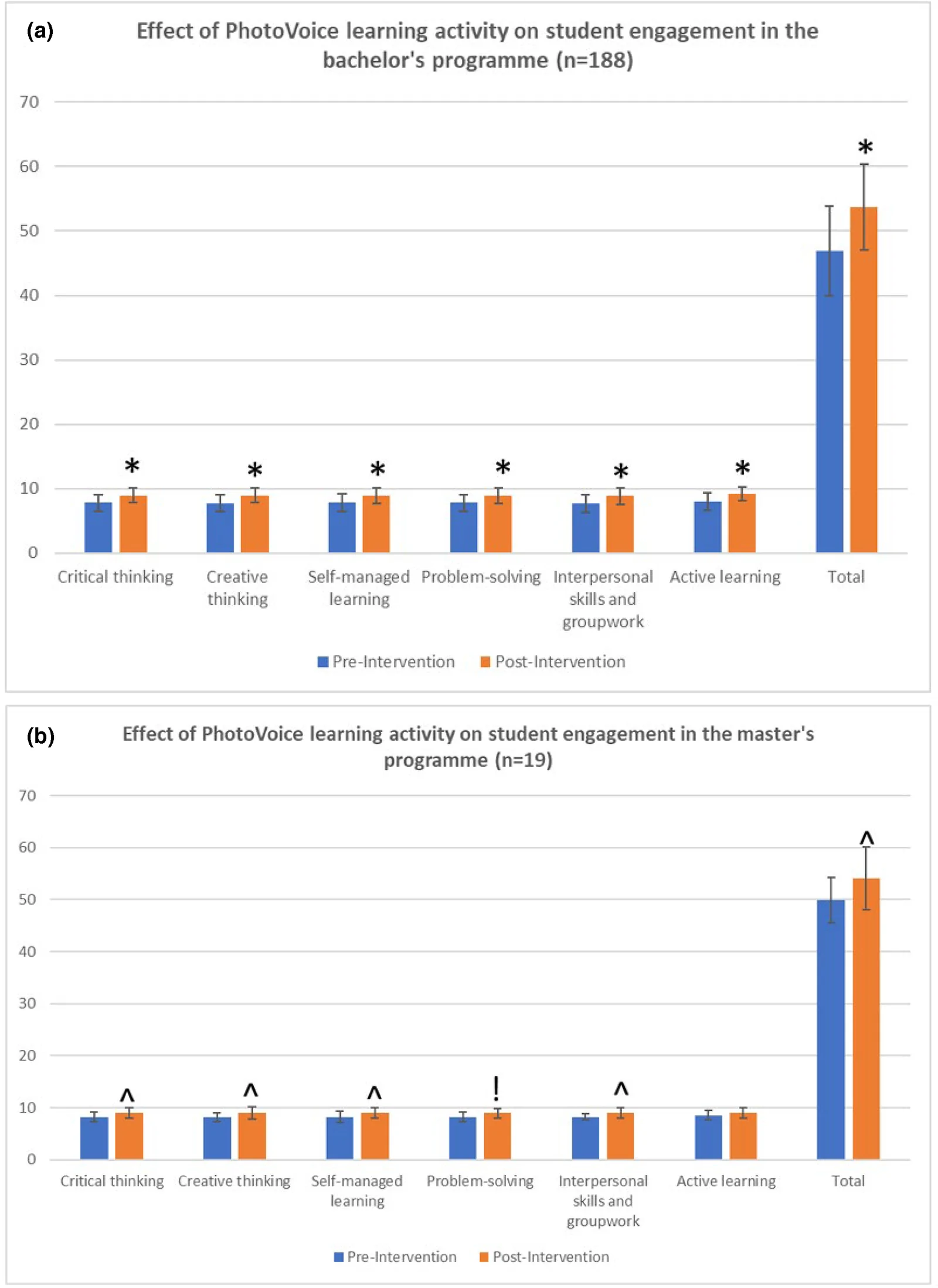
Effect of PhotoVoice learning activity on student engagement. (**a**) Changes in students’ engagement levels in the bachelor’s programme; ^*^ denotes a statistically significant difference between pre- and post-intervention scores (*P*<0.001). (**b**) Changes in students’ engagement levels in the master’s programme (*n*=19); ^^^ denotes a statistically significant difference between pre- and post-intervention scores (*P*<0.01); ^!^ denotes a statistically significant difference between pre- and post-intervention scores (*P*<0.05).

### Student empowerment

Bachelor’s degree students demonstrated significant gains in learning empowerment across all three subscales – impact, meaningfulness and competence – as well as in the total and overall scores. The total empowerment score increased from 33.45 (sd=9.17) to 42.54 (sd=7.94), with a large effect size (Cohen’s *d*=1.08). Among the subscales, the largest effect was observed for impact (Cohen’s *d*=1.06), followed by competence (Cohen’s *d*=0.94) and meaningfulness (Cohen’s *d*=0.91). At the post-intervention assessment, 170 students (90.43%) endorsed the statement *‘I am provided sufficient learning empowerment with the use of the PhotoVoice activity’,* by selecting a rating of three or four on a five-point Likert scale ranging from zero to four.

Master’s degree students similarly reported significant increases in empowerment. The greatest improvement was seen in the impact subscale (Cohen’s *d*=0.96), followed by competence (Cohen’s *d*=0.66) and meaningfulness (Cohen’s *d*=0.63). The total empowerment score increased from 34.21 (sd=8.30) to 40.79 (sd=10.00; Cohen’s *d*=0.84). At the post-intervention assessment, 16 students (84.21%) rated the same empowerment statement with a score of three or four. Table 2 presents the results of student empowerment and Fig. 4 illustrates the comparative changes in empowerment levels before and after the intervention.

**Table 2.** Effect of PhotoVoice learning activity on student empowerment

	Pre-intervention (mean±sd)	Post-intervention (mean±sd)	*P*-value	Cohen’s *d* effect size
**Students in the bachelor’s programme (*n*=188)**				
Impact	12.72±3.97	16.76±3.19	<0.001	1.06
Meaningfulness	12.98±3.46	15.91±3.17	<0.001	0.91
Competence	7.75±2.25	9.87±2.08	<0.001	0.94
Total	33.45±9.17	42.54±7.94	<0.001	1.08
**Students in the master’s programme (*n*=19)**				
Impact	13.00±3.02	16.00±4.08	<0.001	0.96
Meaningfulness	13.05±3.61	15.11±3.93	0.013	0.63
Competence	8.16±2.27	9.68±2.38	0.010	0.66
Total	34.21±8.30	40.79±10.00	0.002	0.84

**Fig. 4.**
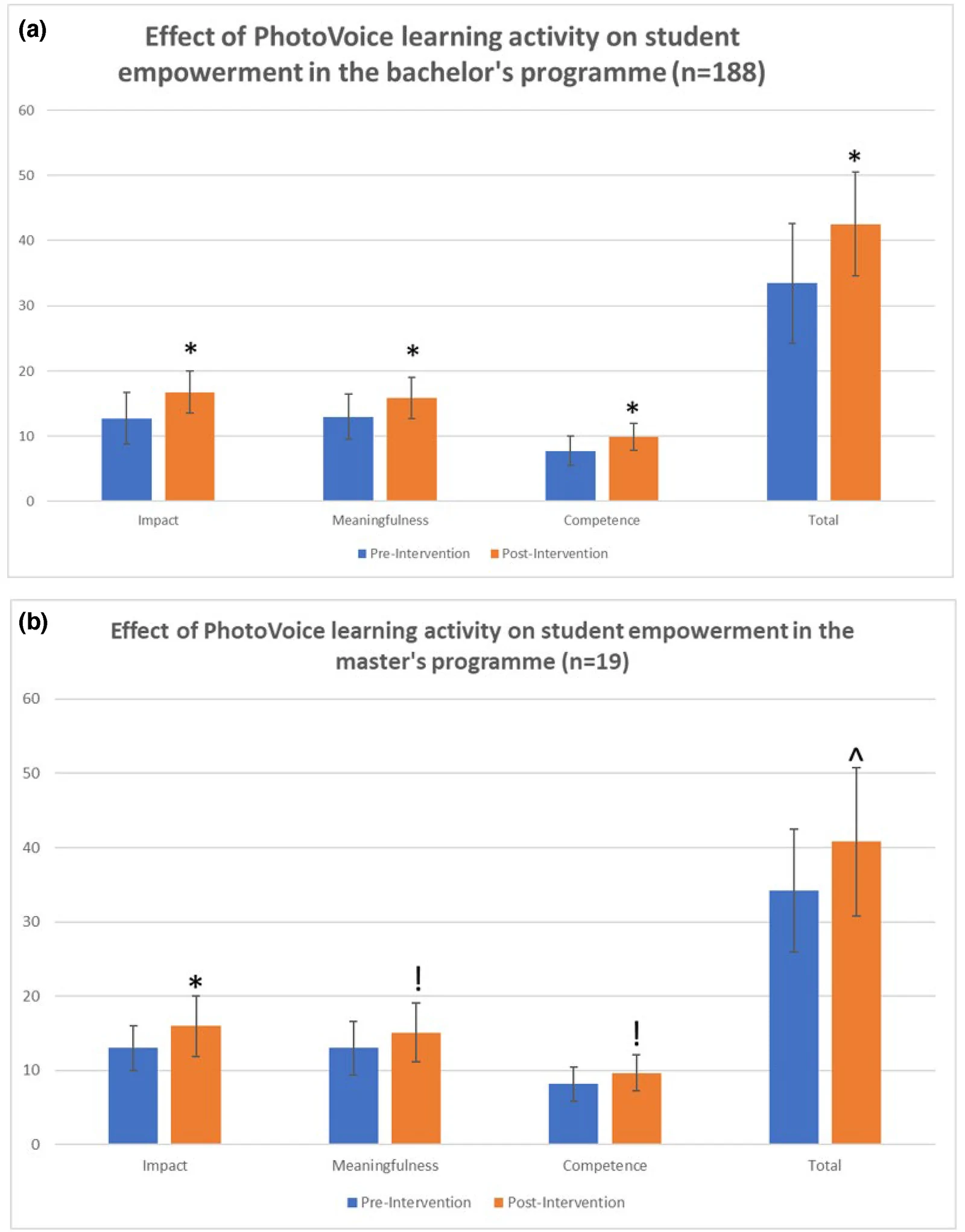
Effect of PhotoVoice learning activity on student engagement. (**a**) Changes in students’ empowerment levels in the bachelor’s programme; ^*^ denotes a statistically significant difference between pre- and post-intervention scores (*P*<0.001). (**b**) Changes in students’ empowerment levels in the master’s programme (*n*=19); ^^^ denotes a statistically significant difference between pre- and post-intervention scores (*P*<0.01); ^!^ denotes a statistically significant difference between pre- and post-intervention scores (*P*<0.05).

### Student satisfaction

In the bachelor’s degree group, student satisfaction statistically significantly increased across all subscales and the total score. The overall satisfaction score rose from 46.82 (sd=6.19) pre-intervention to 51.24 (sd=4.88) post-intervention (Cohen’s *d*=0.75). Among subscales, the largest improvements were noted in generic skills and learning experiences (Cohen’s *d*=0.75), followed by assessment (Cohen’s *d*=0.67) and teaching (Cohen’s *d*=0.61). At the post-intervention assessment, 185 students (98.40%) indicated satisfaction with the quality of the training involving the PhotoVoice activity.

By contrast, no statistically significant changes were observed in satisfaction levels among master’s degree students. Neither the total satisfaction score nor the subscale scores showed statistically significant change post-intervention. The post-intervention total satisfaction score and subscale scores were observed to be lower than those recorded pre-intervention. The sd values of the subscales – Teaching, Generic Skills and Learning Experiences – were higher post-intervention compared to pre-intervention. Nonetheless, 17 students (89.47%) reported being satisfied with the quality of the training involving the PhotoVoice activity. Table 3 presents the results of student satisfaction Fig. 5 illustrates the comparative changes in satisfaction levels before and after the intervention.

**Table 3.** Effect of PhotoVoice learning activity on student satisfaction

	Pre-intervention (mean±sd)	Post-intervention (mean±sd)	*P*-value	Cohen’s *d* effect size
**Students in the bachelor’s programme (*n*=188)**				
Teaching	21.91±2.76	23.55±2.13	<0.001	0.61
Assessment	8.30±1.39	9.21±1.14	<0.001	0.67
Generic skills and learning experiences	16.61±2.56	18.48±2.04	<0.001	0.75
Total	46.82±6.19	51.24±4.88	<0.001	0.75
**Students in the master’s programme (*n*=19)**				
Teaching	23.42±2.29	22.58±4.71	0.445	0.18
Assessment	9.11±1.20	8.95±1.87	0.742	0.08
Generic skills and learning experiences	18.11±1.85	17.84±4.06	0.788	0.06
Total	50.63±4.78	49.37±10.29	0.610	0.12

**Fig. 5.**
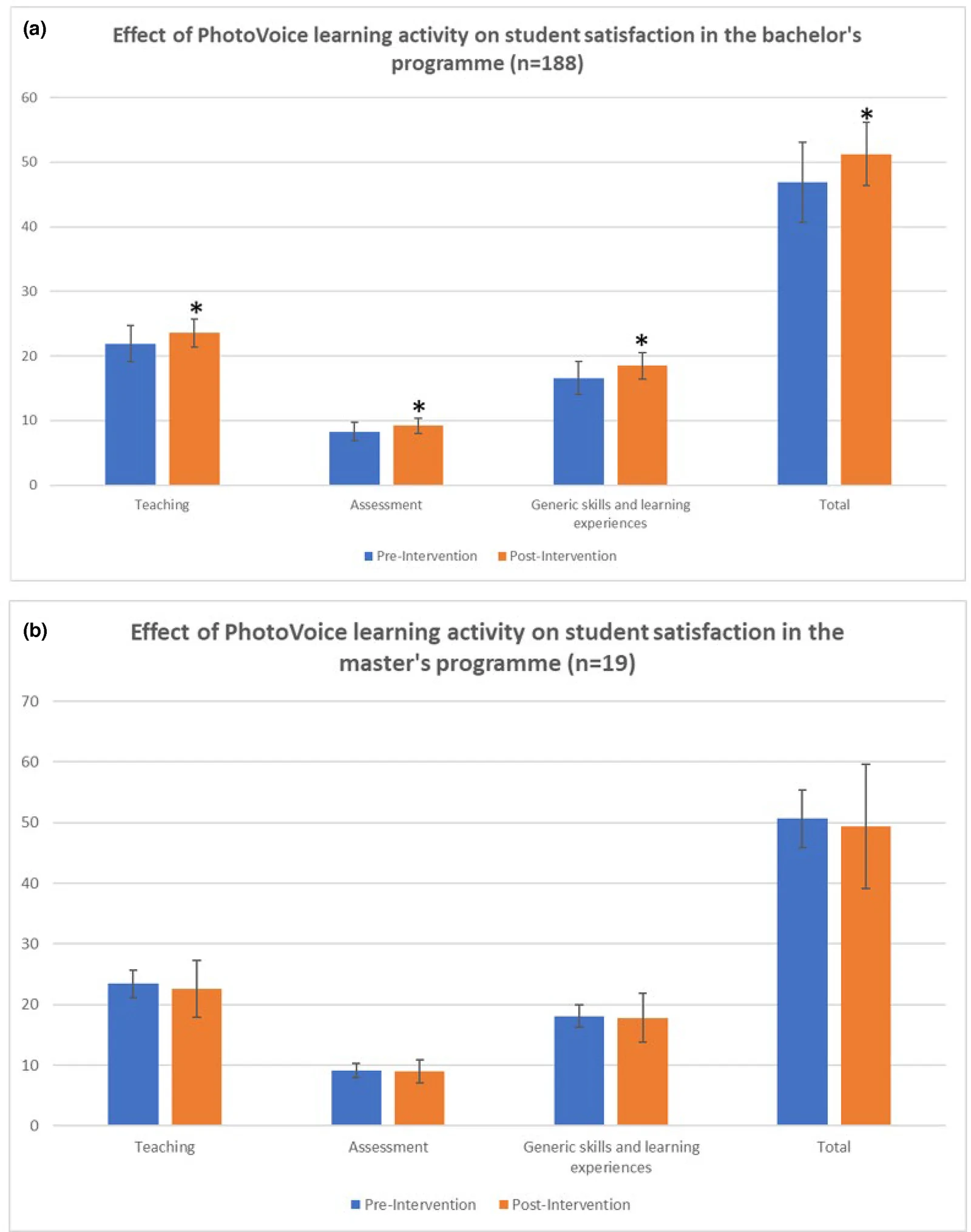
Effect of PhotoVoice learning activity on student engagement. (**a**) Changes in students’ empowerment levels in the bachelor’s programme; ^*^ denotes a statistically significant difference between pre- and post-intervention scores (*P*<0.001). (**b**) Changes in students’ empowerment levels in the master’s programme (*n*=19).

### Qualitative findings: students’ experiences with the PhotoVoice learning activity

In total, 128 nursing students (109 bachelor’s, 19 master’s) participated in group interviews. The inductive content analysis of interview data revealed five key categories reflecting how students experienced the PhotoVoice learning activity: (i) enhanced student engagement, (ii) active and experiential learning, (iii) intellectual stimulation, (iv) authentic learning contexts and (v) innovative pedagogical approach. Each category comprised subcategories that captured distinct but related aspects of student responses. Illustrative quotes are presented to support each theme (Table 4).

**Table 4.** Examples of pre-registration nursing students’ responses in content analysis (both bachelor’s and master’s degree programmes)

Meaning unit	Interpretation	Subcategory	Category	Category
‘PhotoVoice learning activity is very interesting and funny’	The activity stimulates students’ interest and motivation	Increased learning motivation	Enhanced student engagement	Improvement of learning
‘PhotoVoice learning activity provides a good learning environment’	The activity fosters a student-centred and supportive learning context	Positive learning environment		
‘PhotoVoice learning activity allows me to participate more’	The activity encourages active participation in learning	Active participation		
‘I have hands-on experience to rate the cleanliness of inanimate object’	Students are involved in hands-on, exploratory learning	Practical engagement in research	Active and experiential learning	
‘I can find out the answer by myself’	Students develop autonomy and self-directed learning	Learning autonomy		
‘I am impressed by the result’	PhotoVoice learning activity fosters students' intellectual stimulation The activity promotes curiosity and intellectual engagement	Emotional and intellectual impact	Intellectual stimulation	
'PhotoVoice learning activity is applicable and allows me to test the cleanliness of inanimate objects around us’	The task is grounded in real-world, everyday experience	Real-life applicability	Authentic learning contexts	
‘It is my first time to learn microbiology in this way’	The activity introduces an innovative, unfamiliar teaching approach	Novel learning experience	Innovative pedagogical approach	

#### Enhanced student engagement

Students widely described the activity as enjoyable and motivating, noting that it supported their involvement in learning. This pattern was reflected in comments such as: *‘PhotoVoice learning activity is very interesting and funny’,* reflecting heightened learning motivation. Others emphasized the student-centred nature of the experience: representative quotes included ‘PhotoVoice learning activity provides a good learning environment’ and ‘PhotoVoice learning activity allows me to participate more’.

These responses suggest that the activity helped foster a supportive environment, increased motivation and encouraged more active participation in the learning process.

#### Active and experiential learning

This category captures how the activity enabled students to participate actively and apply microbiological concepts through direct experience. Several students noted their involvement in simple observational research, such as: *‘I have hands-on experience to rate the cleanliness of inanimate object’.* Others reflected on the autonomy the activity allowed: *‘I can find out the answer by myself’.* These responses indicate that the activity supported self-directed learning and reinforced practical application through experiential tasks.

#### Intellectual stimulation

Students expressed a sense of intellectual curiosity and surprise as a result of the activity. This pattern was reflected in statements such as: *‘I am impressed by the result’.* Such remarks suggest that the learning activity provoked deeper reflection and critical engagement with microbiological concepts.

#### Authentic learning contexts

Students highlighted the real-life applicability of the learning task, particularly in relation to hygiene and cleanliness in everyday settings. This finding was exemplified by comments such as: *‘PhotoVoice learning activity is applicable and allows me to test the cleanliness of inanimate objects around us’.* This category demonstrates how the activity helped bridge academic knowledge with practical, real-world contexts.

#### Innovative pedagogical approach

Students commented on the novelty of the learning method, viewing it as a refreshing departure from traditional classroom-based teaching. This viewpoint was illustrated in remarks such as: *‘It is my first time to learn microbiology in this way’.* These responses reflect how the PhotoVoice activity offered a new platform for learning that was perceived as more engaging and memorable.

## Discussion

This study demonstrates that integrating a PhotoVoice learning activity into pre-registration nursing microbiology courses is associated with enhanced student engagement and learning empowerment among both undergraduate and master’s pre-registration nursing students. Improvements across all engagement and empowerment subscales for both groups suggest that PhotoVoice offers a viable means of addressing the longstanding challenge of motivating students in microbiology – a subject often perceived as abstract and disconnected from clinical practice.

Engagement in class is a key determinant of students’ academic performance and overall success in higher education [39, 40]. A meta-analysis of 69 independent studies (196,473 participants) revealed that there was a moderately strong and positive correlation between overall student engagement and academic achievement [41]. Engagement facilitates active learning, wherein students process information more deeply through participation, questioning and application. Our results indicate that the PhotoVoice activity is related to enhanced students’ engagement overall, as well as in specific domains including critical thinking, creative thinking, self-managed learning, problem-solving, interpersonal skills and group work and active learning. Thus, interventions that promote engagement – particularly those integrating reflective and collaborative elements like PhotoVoice – can serve as catalysts for deeper learning and skill development in academic settings.

The PhotoVoice approach stimulates creativity by inviting students to identify the cleanest and dirtiest locations on campus, followed by group discussion to select final sites and conduct swab sampling. This process fosters self-managed learning, interpersonal communication and teamwork – skills that are essential in clinical practice [42]. Prior to presenting their findings, students addressed the SHOWED framework, including the question, ‘*What can we do about this problem or situation?*’ This encouraged them to connect real-world contexts with lecture content. Importantly, discrepancies between students’ expectations and the ATP cleanliness data prompted them to explain unexpected results, thereby strengthening their problem-solving and critical thinking skills. Qualitative feedback reinforced these findings, with students describing the activity as ‘interesting’, ‘providing a good learning environment’ and offering ‘hands-on experience to examine the cleanliness of inanimate objects’. Such comments illustrate the value of PhotoVoice in enhancing motivation, creating an authentic, student-centred, research-informed learning environment.

Learning empowerment is also shown to positively influence students’ engagement and academic success in higher education [43, 44], by fostering a sense of responsibility and ownership. Consistent with this, our results showed marked increases in overall learning empowerment and in the subscales of impact, meaningfulness and competence for both academic programmes. The PhotoVoice activity provided students with the freedom and opportunity to learn independently, co-construct microbiology knowledge and relate it to their daily lives in collaboration with peers and lecturers. Students perceived their contributions as meaningful and valued the opportunity to present research-informed findings in class. The hands-on cleanliness testing reinforced their sense of competence as future health professionals capable of identifying potential hygiene issues and proposing evidence-based solutions.

Regarding student satisfaction, we observed a significant improvement among undergraduate students but not among master’s students. The post-intervention total satisfaction score and subscale scores were observed to be lower than those recorded pre-intervention. A likely explanation is the ceiling effect: pre-intervention satisfaction scores for master’s students were already high, with a mean of 50.63 out of a possible 55. The sd values of the subscales – Teaching, Generic Skills and Learning Experiences – were higher post-intervention compared to pre-intervention, indicating varied student experiences following the intervention. This pattern differs from that observed in the bachelor’s cohort. It is possible that the smaller sample size of master’s students contributed to the more pronounced differences in sd values. Nevertheless, 17 master’s students (89.47%) reported being satisfied with the PhotoVoice training post-intervention, indicating positive engagement despite the lack of measurable change. Student satisfaction is an important indicator of teaching quality in higher education, with evidence linking it to improved engagement and academic performance [45, 46]. Based on the group interviews, the activity was described by students as ‘fun’, ‘applicable’ and ‘my first-time learning microbiology in this way’, with others noting, ‘I can find out the answer by myself’ and ‘I am impressed by the results’.

Taken together, the quantitative and qualitative findings demonstrate that PhotoVoice is a practical and engaging pedagogical tool that enhances student engagement, empowerment and – at least among undergraduates – satisfaction. By providing an authentic, interactive and student-centred approach to microbiology, it holds promise for improving motivation and bridging the gap between theoretical content and clinical relevance.

Several limitations should be acknowledged. First, this study employed a non-experimental design. Without a control group, the effectiveness of the photovoice activities on student outcomes cannot be conclusively established. Future research employing more rigorous designs, such as randomized controlled trials, is warranted. Second, all outcomes were self-reported via questionnaires, which may be subject to various biases, including social desirability bias. Future studies could consider incorporating more objective outcome measures, such as examination results. Finally, as this was a single-site study, the generalizability of the findings may be limited.

## Conclusion

This study provides evidence that integrating a PhotoVoice learning activity into pre-registration nursing microbiology courses is associated with enhanced student engagement, learning empowerment and, among undergraduates, satisfaction. The PhotoVoice approach, characterized by its participatory, experiential and student-centred nature, effectively bridges theoretical microbiological knowledge with real-world clinical relevance. Students reported heightened motivation, deeper critical thinking, improved teamwork and greater empowerment, highlighting the pedagogical value of this innovative method. While the findings are promising, future research with more rigorous designs and objective outcome measures is needed to further establish the effectiveness and generalizability of PhotoVoice in nursing education. Incorporating active, authentic learning strategies like PhotoVoice may contribute meaningfully to closing knowledge gaps and better preparing nursing students for the complexities of infection prevention and clinical practice.

## Supplementary material

10.1099/acmi.0.001265.v3 Supplementary Material 1.

10.1099/acmi.0.001265.v3 Supplementary Material 2.

## Abbreviations

ATP: adenosine triphosphate
